# Effects of a ready-to-drink thermogenic beverage on resting energy expenditure, hemodynamic function, and subjective outcomes

**DOI:** 10.1080/15502783.2023.2211958

**Published:** 2023-05-10

**Authors:** Christian Rodriguez, Matthew T. Stratton, Patrick S. Harty, Madelin R. Siedler, Jake R. Boykin, Jacob J. Green, Dale S. Keith, Sarah J. White, Brielle DeHaven, Alexandra Brojanac, Ethan Tinoco, Lem W. Taylor, Grant M. Tinsley

**Affiliations:** a Texas Tech University, Energy Balance & Body Composition Laboratory; Department of Kinesiology & Sport Management, Lubbock, TX, USA; b University of Mary Hardin-Baylor, Human Performance Laboratory, School of Exercise and Sport Science, Belton, TX, USA

**Keywords:** Caffeine, metabolism, stimulant, weight loss, energy drink

## Abstract

**Background:**

Thermogenic supplements are often consumed by individuals seeking to improve energy levels and reduce body fat. These supplements are sold in powdered or ready-to-drink (RTD) forms and consist of a blend of ingredients such as caffeine, green tea extract, and other botanical compounds. While there is evidence that thermogenic supplements can positively affect resting energy expenditure (REE), the effect varies based on the combination of active ingredients. Additionally, there is some concern that thermogenic supplements may cause unwanted side effects on hemodynamic variables, like heart rate (HR) and blood pressure (BP). Therefore, further investigation into the efficacy and safety of commercially available products is warranted.

**Methods:**

Twenty-eight individuals (14 F, 14 M; age: 23.3 ± 3.9 yrs; height: 169.4 ± 8.6 cm; body mass: 73.3 ± 13.1 kg) completed two visits in a randomized, double-blind, crossover fashion. Each visit began with baseline REE, HR, and BP assessments, which were followed by ingestion of an active RTD thermogenic beverage (RTD; OxyShred Ultra Energy) or placebo (PL). Assessments were repeated at the intervals of 35–50- and 85–100-minutes post-ingestion. In addition, subjective outcomes of energy, focus, concentration, alertness, and mood were collected five times throughout each visit. Repeated-measures analysis of variance was performed with condition and time specified as within-subjects factors and sex and resistance training (RT) status as between-subjects factors. Statistical significance was accepted at *p* < 0.05.

**Results:**

A significant condition × time interaction was observed for REE (*p* < 0.001). Higher REE values were demonstrated at 35–50 min (0.08 ± 0.02 kcal/min; *p* = 0.001; 5.2% difference) and 85–100 min (0.08 ± 0.02 kcal/min; *p* = 0.001; 5.5% difference) after RTD ingestion as compared to PL. No significant condition × time interactions were observed for respiratory quotient, HR, or BP. Condition main effects indicated lower HR (3.0 ± 0.9 bpm; *p* = 0.003), higher SBP (3.5 ± 1.1 mm Hg; *p* = 0.003) and higher DBP (3.5 ± 0.9 mm Hg; *p* < 0.001) in RTD as compared to PL, irrespective of time. Condition × time interactions were observed for all subjective outcomes (*p* ≤ 0.02). Post hoc tests indicated statistically significant benefits of the RTD over PL for energy, focus, concentration, and alertness, without significant differences for mood after correction for multiple comparisons. Sex and RT status were not involved in interactions for any outcomes, except for a Sex × RT status interaction for energy, indicating higher energy ratings in non-resistance-trained vs. resistance-trained males.

**Conclusions:**

These data suggest that acute ingestion of a thermogenic RTD beverage significantly increases REE, and this elevated caloric expenditure is sustained for at least 100 minutes following ingestion. Furthermore, the RTD beverage increased measures of energy, focus, concentration, and alertness as compared to placebo. While minor differences in hemodynamic variables were observed between conditions, all values stayed within normal ranges. Individuals aiming to increase energy expenditure may benefit from acute ingestion of an RTD thermogenic supplement.

## 1. Introduction

The obesity epidemic continues to surge worldwide, and there are few indications of it slowing. Given the current trends, it is projected that 1 in 2 U.S. adults will have obesity by 2030, and 1 in 4 adults will have severe obesity by 2030 [1]. While a variety of therapies are available to assist in combatting obesity, this epidemic has caused many individuals who are overweight or obese to turn to dietary supplements and related products, such as energy drinks, for additional assistance [2,3]. Indeed, among all U.S. adults from 2007 through 2018, age-adjusted dietary supplement use increased from 48.4% to 56.1% [4]. Additionally, energy drink consumption increased from 2003 to 2016 in adolescents (0.2% to 1.4%, *p* = 0.028), young adults (0.5% to 5.5%, *p* < 0.001), and middle-aged adults (0.0% to 1.2%, *p* = 0.006) [5]. With the prevalence of supplement and energy drink use on a steady climb, and different forms of oversight by regulatory bodies over these products relative to pharmaceuticals, clinical trials evaluating the safety and efficacy of popular products are needed [6]. Dietary supplements are sold for a variety of purposes, and while consumers have various motives for consuming them, augmented weight loss and energy are two previously documented reasons [2]. A popular class of products, termed thermogenics, are formulated to target these outcomes. Thermogenics can be defined as products designed to increase thermogenesis and energy expenditure. These products may target multiple mechanisms for weight and fat loss including increasing resting energy expenditure (REE), promoting lipolysis, and modulating appetite and hunger [7,8]. Thermogenics often contain ingredients that may upregulate uncoupling of the respiratory chain in mitochondria and potentiate thermogenesis either in conjunction with stimulating the central nervous system (CNS) (stimulant thermogenics) or with no significant stimulation of the CNS (non-stimulant thermogenics) [9]. Common signs of CNS stimulatory activity are increases in heart rate (HR) and blood pressure (BP) [9]. Given the purported effects of thermogenic products, it is common for consumers to purchase these products anticipating that they will increase caloric expenditure, lipolysis, and assist in weight management [10]. Thermogenic products are sold in a multitude of forms including pills, powder, or ready-to-drink (RTD) forms, the latter of which may be considered a dietary supplement or a conventional food item (i.e. beverage) depending on labeling, recommended use, and other factors [11]. Regardless of form, these products typically consist of a blend of ingredients such as caffeine, green tea extract, and other herbal compounds. Caffeine functions through inhibition of phosphodiesterase and antagonizing adenosine receptors, leading to accumulation of intracellular 3,5-cyclic-adenosine monophosphate (cAMP), which is metabolically excitatory for cells [2]. However, combining caffeine with other active ingredients such as green tea extract appears to be a more efficacious option for producing a thermogenic effect on REE and fat oxidation over that of caffeine alone [2,12,13]. Indeed, previous data indicate that commercially available multi-ingredient thermogenic supplements potentiate increases in REE when ingested acutely and may favorably affect body composition estimates chronically [7,8,12–17]. Furthermore, mixed findings have been noted regarding the effects of training status on the metabolic response to caffeine, and Harty et al. [18] proposed that resistance-trained (RT) males and females may respond differently to acute caffeine supplementation due to potential differences in body size, composition, and hormonal makeup [19,20]. Further research is needed to clarify the potential influences sex and training status has on the metabolic response to acute caffeine consumption. Additionally, these supplements have also been reported to influence subjective measures such as energy, focus, alertness, and mood [7,12,21]. While some evidence suggests thermogenic products are effective for augmenting caloric expenditure, a common concern is their effect on hemodynamic variables, such as HR and BP. For instance, typical side effects associated with caffeine ingestion are tachycardia, heart palpitations, anxiety, and headaches [21]. Though some have reported ingestion of caffeine plus other herbal ingredients (such as guarana, green tea extract, yerba mate, and yohimbine) generates increases in HR and systolic BP (SBP), others have demonstrated no detrimental effects of thermogenic supplements on hemodynamic function [8,12,13,16,22]. Nonetheless, a concern associated with thermogenic products is potentially insufficient scientific evidence regarding their safety and efficacy [23]. Because of their convenience and suggested benefits, these products may be more susceptible to misuse when individuals consume more than the recommended quantities [24]. The combination of multiple ingredients, concentrated amounts of caffeine, and potentially excessive consumption of these products may increase the likelihood of adverse effects [24]. Although energy products, weight loss products, and pre-workout supplements are among the categories of dietary supplements associated with a higher relative risk for adverse effects [24], substantial differences in formulations necessitate evaluation of these products on a case-by-case basis. Accordingly, it is imperative randomized controlled trials be conducted on new formulations of these products. Therefore, the purpose of this study was to determine the acute effects of an RTD thermogenic beverage on REE, respiratory quotient (RQ), hemodynamic variables, and subjective ratings of energy, focus, concentration, alertness, and mood. It was hypothesized that the RTD beverage would elevate REE and improve subjective measures without adverse effects on hemodynamic variables.

## 2. Materials and methods

### 2.1. Overview

This investigation utilized a randomized, double-blind, placebo-controlled crossover design to examine the effects of acute consumption of an RTD thermogenic beverage on REE, RQ, hemodynamic variables, and subjective measures of energy, focus, concentration, alertness, and mood. Each participant completed two laboratory visits. Each visit began with baseline REE, HR, and BP assessments, which were followed by ingestion of an active RTD thermogenic beverage (RTD; OxyShred Ultra Energy, EHP Labs Inc., Sydney, Australia) or placebo (PL; consisting of the flavoring of RTD without the active ingredients). Assessments were repeated at the intervals of 35–50- and 85–100-minutes post-ingestion. In addition, self-reported measures of energy, focus, concentration, alertness, and mood were collected via visual analog scales (VAS) five times throughout each visit: upon arrival (Pre1), 50 minutes following arrival (Pre2), immediately following beverage consumption (Post1), 50 minutes following consumption (Post2), and 100 minutes following consumption (Post3). Visits were scheduled 1 to 10 days apart and effort was made for each participant’s time of day to be repeated for each of their testing visits. All visits were commenced between the hours of 6 AM and 8AM. An overview of the study’s procedures is displayed in Table 1. The experimental protocol was granted ethical approval by Texas Tech University’s Institutional Review Board (Protocol #: 2021–676; date of approval: 10/1/2021), and all participants provided written informed consent before participation. In addition, the study was prospectively registered on ClinicalTrials.gov (Identifier: NCT05194475).

**Table 1.** Overview of study visit procedures.

Procedure		Time (min)^1^
1^st^ Interval	Supine Rest	30.1 ± 0.7
	Hemodynamic Assessments	3.5 ± 0.8
	Indirect Calorimetry Test	22.0 ± 1.0
	Beverage Consumption	14.8 ± 1.1
2^nd^ Interval	Supine Rest	29.5 ± 4.0
	Hemodynamic Assessments	3.7 ± 4.2
	Indirect Calorimetry Test	22.1 ± 0.3
3^rd^ Interval	Supine Rest	30.1 ± 0.3
	Hemodynamic Assessments	3.2 ± 1.4
	Indirect Calorimetry Test	21.9 ± 1.4

Data are presented as mean ± SD.

^1^These times represent the duration from the start of the listed procedure to the next procedure.

### 2.2. Participants

The target population for this investigation was RT and non-resistance-trained (NRT) males and females between 18 and 40 years of age. Participants had to have a body mass between 50 to 100 kg (110 to 220 pounds) in order to truncate the range of relative caffeine doses (i.e., the dose of caffeine in mg per kg of body mass). The purpose of this restriction was to promote a relatively consistent dose/intervention within our sample. RT was defined as performing ≥ 3 RT sessions per week for at least 2 years prior to screening. Additionally, the entire body (all major upper body and lower body muscle groups) must have been trained at least once weekly. NRT was defined as having never consistently followed a structured RT program. To be eligible, at the time of screening, all participants could not be currently performing>30 minutes of high-intensity interval training (HIIT) per week or >60 minutes of low-intensity steady-state endurance exercise per week. This was due to prior data demonstrating a potential effect of endurance exercise training status on the metabolic response to caffeine ingestion [19,20] and a desire to examine RT status as a potential influencer in this regard. Participants also had to regularly consume caffeine (due to the presence of caffeine in the commercially available supplement). Habitual caffeine consumption was defined as an average self-reported daily intake of ≥200 mg of caffeine, or approximately 2 cups of coffee. Participants were ineligible if they had a presence of any known disease or medical condition which could be negatively affected by consumption of the beverage, were pregnant or breastfeeding, were taking medication which could reasonably make participation unsafe for the participant or influence study outcomes, had a self-reported caffeine sensitivity, were allergic to any of the ingredients in the RTD beverage, had a pacemaker or other implanted electrical device, or had self-reported claustrophobia (due to metabolism testing). Interested individuals meeting these criteria completed the consent process before continuing with the study. Additionally, female participants were asked to fill out a brief document related to their menstrual cycle history and contraceptive use to allow for a more complete description of our sample.

Twenty-eight college-aged males (*n* = 14) and females (*n* = 14) volunteered to participate in this study. Within each group of males and females, seven were RT and seven were NRT. Participant characteristics are displayed in Table 2. Additionally, participant characteristics split by sex only and RT status only are presented in **Supplementary Material 1**. Of the 14 females who participated in the study, eleven (~79%) reported having a regular menstrual cycle, defined as menstrual periods that occur at predictable intervals and no missed periods in the past six months, seven (50%) reported being on some form of hormonal contraception. Of these, four (~29%) reported using a combined oral contraceptive pill, two (~14%) reported using a progestin-only oral contraceptive pill, one (~7%) reported using a hormonal intrauterine device.

**Table 2.** Participant Characteristics.

	All (*n* = 28)		F NRT (*n* = 7)		F RT (*n* = 7)		M NRT (*n* = 7)		M RT (*n* = 7)	
	mean	sd	mean	sd	mean	Sd	mean	sd	mean	Sd
Age (y)	23.3	3.9	20.9	1.8	23.7	3.5	24.6	4.8	24.0	4.4
Height (cm)	169.3	8.6	164.0	3.6	163.3	7.1	176.6	7.2	173.5	8.0
Weight (kg)	73.3	13.1	61.5	8.3	64.2	8.2	83.1	7.5	84.5	7.4
BMI (kg/m^2^)	25.5	3.3	22.9	3.2	24.1	2.7	26.7	2.6	28.1	2.3
FFMI (kg/m^2^)	18.6	2.5	16.0	1.4	17.2	0.7	19.9	1.1	21.4	2.0
Body fat (%)	26.7	5.7	29.5	4.6	28.1	5.4	25.1	7.1	24.0	4.8
RT Experience (years)	2.6	3.8	0.0	0.0	3.2	0.8	0.0	0.0	7.4	4.5
RT Frequency (days/week)	2.3	2.4	0.0	0.0	4.6	0.5	0.0	0.0	4.5	1.2
Caffeine Intake (mg/d)	302.0	117.6	235.0	40.7	235.7	55.6	301.4	107.3	435.7	121.5
Phase Angle (degrees)	6.0	0.8	5.3	0.4	5.6	0.6	6.3	0.5	6.8	0.8
Waist Circumference (cm)	90.3	6.8	88.1	8.3	87.9	7.7	94.4	5.1	90.9	4.9
Hip Circumference (cm)	105.3	5.5	103.4	6.5	102.5	4.1	106.9	5.4	108.4	4.3
Upper Arm Circumference (cm)	35.4	4.0	32.2	3.4	32.9	3.2	36.9	2.0	39.5	2.6
Forearm Circumference (cm)	27.8	2.6	25.1	1.6	26.1	1.6	29.5	0.7	30.5	0.6
Thigh Circumference (cm)	61.0	4.5	58.5	5.4	60.0	4.3	61.6	3.7	63.7	3.6
Calf Circumference (cm)	38.1	2.8	36.2	3.8	37.0	1.1	40.2	1.8	39.2	2.0

*participant characteristics split by sex only and RT status only are presented in Supplementary Material 1*.

*Abbreviations*: BMI, body mass index; F, females; FFMI, fat-free mass index; M, males; NRT, non-resistance trained; RT, resistance training.

### 2.3. Testing procedures

For each testing session, participants reported to the Energy Balance & Body Composition Laboratory in the morning after abstaining from food, caffeine, alcohol, nicotine, supplements, and medication for at least eight hours prior to their visit. In addition, they were asked to refrain from exercising or engaging in vigorous physical activity the day prior and the morning of the visit. Participants were also instructed to use the same method of transportation for both visits. Participants were then interviewed to confirm adherence to these pre-assessment instructions. Upon voiding their bladder, each participant’s height was determined using a mechanical stadiometer (HM200P, Charder Medical), and body mass was measured using a digital scale (Seca 769, Hamburg, Germany). For descriptive purposes, anthropometric and body composition assessments were then conducted on the first visit only. Participants were first given a swim cap to cover all hair and prevent artifacts from influencing the test, then anthropometric values were collected using a three-dimensional optical scanner (SS20, Size Stream, scanner version 6.2, software version 5.2.7 for Size Stream Studio). Body composition was estimated using multi-frequency bioelectrical impedance analysis (Seca mBCA 515/514, Seca, Hamburg, Germany).

Prior to the baseline REE assessment, participants lay supine for a period of 30 minutes. This 30-minute supine rest period preceded each REE assessment. After each 30-minute supine rest period and immediately before each REE assessment, HR, SBP, and diastolic BP (DBP) values were recorded utilizing an automated, portable, digital BP monitor (Omron BP 785). REE was then assessed via indirect calorimetry (TrueOne 2400, ParvoMedics, Sandy, UT, USA). Gas and flow calibrations were performed each morning according to manufacturer specifications. Pre-assessment and procedural standardization were based on the recommendations of Fullmer et al. [25]. Testing took place in a climate-controlled room with the lights dimmed. The room temperature was maintained between 22 and 25ºC (i.e. 72 to 77ºF), and if the temperature fell below 22ºC, a blanket was provided to the participant. The participant could also choose to use a blanket even if the temperature was within the 22 to 25ºC range, but if chosen, the blanket was used for all REE tests in both of their study visits. For each REE assessment, participants rested in a supine position for 20 minutes while expired air was collected and analyzed. Participants were instructed to remain motionless but awake during the assessment. Reported data represent a 15-minute average after discarding the first 5 minutes of each REE assessment. REE and hemodynamic assessments took place at baseline and 35–50- and 85–100-minutes post supplement-ingestion. Participants were allowed to use the restroom following the completion of each REE assessment. The time-point(s) at which each participant did or did not use the restroom was kept standardized between their two study visits.

### 2.4. Beverage ingestion

Beverage ingestion took place immediately after the baseline REE assessment. Participants consumed two refrigerated (4°C) 12-fluid ounce (355 ml) cans of either the active RTD thermogenic beverage or PL each study visit. This dose was selected due to manufacturer instructions, which recommend consuming one to two cans for healthy individuals. The RTD beverage contained 180 mg of caffeine per serving (1 can) coming from a blend of caffeine anhydrous, green tea extract, and guarana. Thus, each participant consumed 360 mg of caffeine during the RTD study condition, which is slightly lower than 400 mg, an amount the United States Food & Drug Administration (FDA) has cited as a total daily caffeine intake not typically associated with adverse effects [26]. RTD and PL were both provided by the study sponsor (EHP Labs Inc., Sydney, Australia). The nutrition facts for the RTD beverage are displayed in Table 3, along with the PL ingredients. PL was a flavor-matched beverage containing all the ingredients present in RTD except for the active ingredients. The order of beverage ingestion was determined using *randomizeR* package and a randomized permuted block design (*pbrPar* function), with blocks for RT males, NRT males, RT females, and NRT females. Condition sequences were randomized and balanced within each block. To facilitate participant blinding, the previously refrigerated beverages were emptied into an opaque shaker bottle by the lone unblinded investigator before being provided to participants for ingestion. Participants were given a total of 15 minutes to drink both servings of the assigned beverage, which consisted of 7.5-minute time intervals in which they were to consume each serving. Additionally, participants were instructed to refrain from commenting on the supplement’s flavor, texture, or how it made them feel. At the end of each visit, participants were asked to report any side effects they experienced throughout the visit. To assess blinding efficacy, participants received a questionnaire at the end of each visit in which they were to report whether they believed they received the RTD or PL condition. These questionnaires were only viewed by the unblinded investigator who prepared the supplemental conditions.

**Table 3.** Nutrition facts and ingredients for ready-to-drink beverage^1^.

Serving Size 12 fluid ounces (355 ml)	
**Amount Per Serving**	
**Calories**	**0**
	**% Daily Value**
Total Fat 0 g	0%
Cholesterol 0 mg	0%
Sodium 20 mg	1%
Total Carbohydrate<1 g	0%
Dietary Fiber 0 g	0%
Total Sugars 0 g	0%
Protein 0 g	
Vitamin D 0mcg	0%
Calcium 10 mg	0%
Iron 0 mg	0%
Potassium 20 mg	0%
Vitamin C 50 mg	60%
Thiamin (B1) 4 mg	330%
Riboflavin (B2) 2 mg	150%
Niacin (B3) 40 mg	250%
Vitamin B6 1 mg	60%
Vitamin B12 10mcg	420%
Pantothenic Acid (B5) 2 mg	40%
Chromium 50mcg	140%^a^

Ingredients: Filtered Sparkling Carbonated Water, L-Carnitine Tartrate, Natural Flavor, Citric Acid, Taurine, Malic Acid, Fumaric Acid, Sucralose, Caffeine, N-Acetyl L-Tyrosine, Inositol, Sodium Citrate, Potassium Sorbate, Green Tea Extract, Ascorbic Acid (Vitamin C), Nicotinamide (Vitamin B3), Guar Gum, Caramel Color, Guarana Seed Extract, Chromium Picolinate, Thiamine Hydrochloride (Vitamin B1), Riboflavin (Vitamin B2), Calcium Pantothenate (Vitamin B5), Pyridoxine Hydrochloride (Vitamin B6), Methylcobalamin (Vitamin B12).

Total Caffeine (from Caffeine Anhydrous + Green Tea Extract + Guarana)180 mg per serving.

^a^Ingredients for placebo beverage included: Carbonated water, Potassium sorbate, Trisodium citrate dihydrate, sucralose, fumaric acid, citric acid, malic acid, ascorbic acid, guar gum, natural pina colada flavor, and natural pineapple flavor. These ingredients were present in the same quantities found in the ready-to-drink beverage.

### 2.5. Subjective measures

VAS for subjective measures of energy, focus, concentration, alertness, and mood were collected five times throughout each visit: 1) Upon arrival, prior to lying down for the first 30-minute time interval (Pre1); 2) Immediately post-baseline REE assessment (Pre2); 3) Immediately post supplement-ingestion (Post1); 4) Immediately following the second REE assessment at~50-minutes post-ingestion (Post2); and 5) Upon completion of the final REE assessment at ~100 minutes post-ingestion (Post3). Similar VAS-derived subjective outcomes were recorded in a recent investigation evaluating the effects of a thermogenic supplement (OxyShred Thermogenic Fat Burner, EHP Labs, Salt Lake City, Utah, USA) on REE, hemodynamic variables, and subjective mood states [27]. Data were self-reported and collected via a software application (VasQ, Maki Nakata) on an electronic tablet. All VAS were grounded with relevant phrases like “extremely low” or “the worst it has ever been” on the far-left side of the scale and a phrase like “extremely high” or “the best it has ever been” on the far-right side of the scale. All values were expressed as a score ranging from 0 to 100, with 0 being the minimum score and 100 being the maximal score.

### 2.6. Statistical analysis

Data were analyzed using R (v. 4.1.2). A repeated-measures analysis of variance (ANOVA) test was performed for each outcome using the *afex* R package [28] with condition and time specified as within-subjects factors and sex and RT status as between-subjects factors. Normality of residuals was examined by visual inspection of quantile-quantile plots, and the Greenhouse-Geisser correction was applied [29]. Follow-up for significant effects was performed using pairwise comparisons with Benjamini & Hochberg adjustment [30] via the *emmeans* R package [31]. Statistical significance was accepted at *p* < 0.05.

## 3. Results

Full ANOVA results for all outcome variables are displayed in **Supplementary Material 2**.

### 3.1. Metabolism

**Figure 1.**
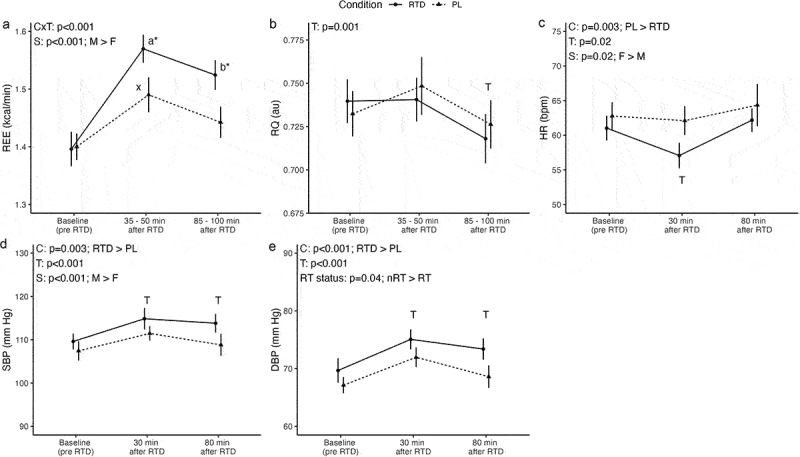
Metabolic and hemodynamic outcomes. Letters within a condition (i.e. a, b, c for the RTD condition and x, y, z for the PL condition) indicate differences relative to the baseline assessment within that condition, with different letters indicating significantly different points. Asterisks indicate a significant difference between conditions at a given time point. Error bars are within-subjects error bars due to the design of the study. RTD: ready-to-drink beverage; PL: placebo beverage; REE: resting energy expenditure; RQ: respiratory quotient; HR: heart rate; SBP: systolic blood pressure; DBP: diastolic blood pressure; CxT: condition × time interaction; S: sex main effect; F: female; M: male; T: time main effect; RT status: RT main effect; RT: resistance-trained; nRT: nonresistance-trained.

A significant condition × time interaction was observed for REE (*p* < 0.001; Figure 1A), but no interactions involving sex or RT status **(Supplementary Material 2)**. Pairwise comparisons indicated no difference in baseline REE ([mean ± SE] 0.0 ± 0.02 kcal/min; *p* = 0.82; 0.3% difference) but higher REE values at 35–50 min (0.08 ± 0.02 kcal/min; *p* = 0.001; 5.2% difference) and 85–100 min (0.08 ± 0.02 kcal/min; *p* = 0.001; 5.5% difference) after RTD ingestion as compared to PL. A sex main effect indicated higher REE values in males as compared to females (0.42 ± 0.05 kcal/min; *p* < 0.001)

For RQ, the sole significant effect was a time main effect (*p* = 0.001), with follow-up pairwise comparisons indicating lower RQ values at 85–100 min after beverage ingestion as compared to baseline (−0.01 ± 0.005 au; *p* = 0.03) and 35–50 min (−0.02 ± 0.006 au; *p* = 0.004), irrespective of condition (Figure 1b).

### 3.2. Hemodynamics

No significant interactions were present for HR, but main effects of condition (*p* = 0.003), time (*p* = 0.02), and sex (*p* = 0.02) were observed (Figure 1c). Follow-up for the condition main effect indicated higher HR in PL as compared to RTD (3.0 ± 0.9 bpm; *p* = 0.003). Follow-up for the time main effect indicated lower HR at 35–50 min after ingestion as compared to baseline (−2.3 ± 0.8 bpm; *p* = 0.02) and 85–100 min (−3.7 ± 1.3 bpm; *p* = 0.02). Finally, follow-up for the sex main effect indicated higher HR in females as compared to males (8.3 ± 3.2 bpm; *p* = 0.02).

Similar to HR, main effects of condition (*p* = 0.003), time (*p* < 0.001), and sex (*p* < 0.001) – but no interactions – were observed for SBP (Figure 1d). Follow-up indicated higher SBP in RTD as compared to PL (3.5 ± 1.1 mm Hg; *p* = 0.003), irrespective of time. Follow-up for the time main effect indicated higher SBP at 35–50 (4.6 ± 1.1 mm Hg; *p* = 0.001) and 85–100 min (2.8 ± 1.1 mm Hg; *p* = 0.02) after beverage ingestion, irrespective of condition. Finally, follow-up to the sex main effect indicated higher SBP in males as compared to females (10.7 ± 2.5 mmHg; *p* < 0.001).

For DBP, no significant interactions were observed. However, main effects of condition (*p* < 0.001), time (*p* < 0.001), and RT status (*p* = 0.04) were present (Figure 1e). Follow-up to the condition main effect indicated higher DBP in RTD as compared to PL (3.5 ± 0.9 mm Hg; *p* < 0.001), irrespective of time. Follow-up to the time main effect indicated higher DBP at 35–50 (5.1 ± 1.0 mm Hg; *p* < 0.001) and 85–100 min (2.6 ± 0.8 mm Hg; *p* = 0.006) after beverage ingestion, as compared to baseline. Finally, follow-up to the RT status main effect revealed that DBP was higher in nonresistance-trained individuals as compared to resistance-trained (4.2 ± 1.9 mmHg; *p* = 0.04).

### 3.3. Subjective variables

**Figure 2.**
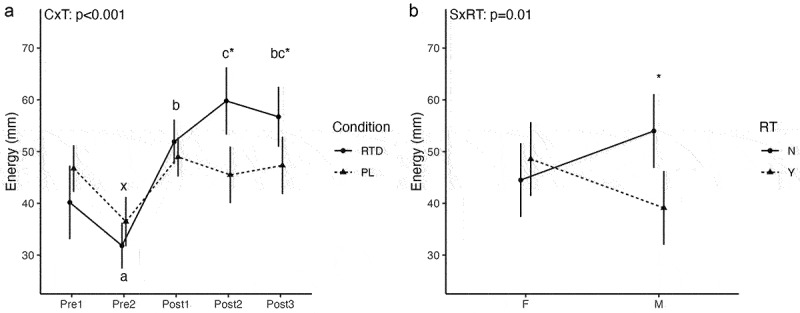
Subjective Energy. Letters within a condition (i.e. a, b, c for the RTD condition and x, y, z for the PL condition) indicate differences relative to the baseline assessment within that condition, with different letters indicating significantly different points. Asterisks indicate a significant difference between conditions at a given time point. Error bars are within-subjects error bars for panel a and model error bars for panel B. CxT: condition × time interaction; SxRT: sex × resistance training status interaction. RT: resistance training status (Y=resistance-trained; N=nonresistance-trained); F: female; M: male.

For energy, statistically significant condition × time (*p* < 0.001) and sex × RT status (*p* = 0.01) interactions were observed. Follow-up for the condition × time interaction revealed that, in the RTD condition, energy was lower at Pre2 (*p* = 0.02; 23% difference) and higher at Post1 (*p* = 0.01; 25% difference), Post2 (*p* = 0.003; 39% difference), and Post3 (*p* = 0.004; 34% difference) as compared to Pre1 (Figure 2a). In PL, energy decreased from Pre1 to Pre2 (*p* = 0.003; 25% difference) but did not differ from Pre1 at other time points. Energy ratings did not differ between conditions at Pre1 (*p* = 0.13), Pre2 (*p* = 0.17), or Post1 (*p* = 0.42), but were higher in RTD than PL at Post2 (*p* = 0.008; 27% difference) and Post3 (*p* = 0.007; 18% difference). Follow-up for the sex × RT status interaction indicated that energy ratings were higher in male non-resistance-trained participants as compared to male resistance-trained participants (*p* = 0.03), without other differences (Figure 2b).

**Figure 3.**
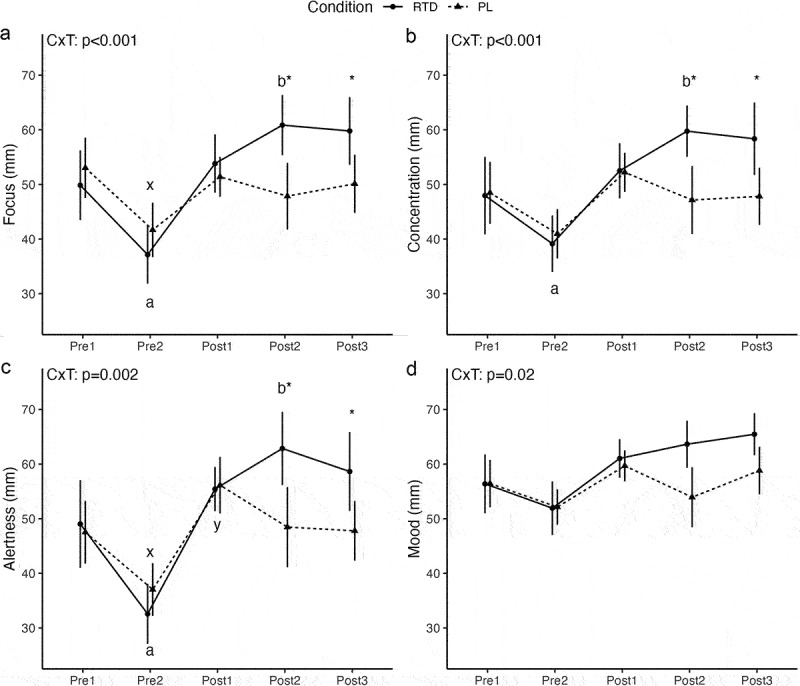
Subjective focus, concentration, alertness, and mood. Letters within a condition (i.e. a, b, c for the RTD condition and x, y, z for the PL condition) indicate differences relative to the baseline assessment within that condition, with different letters indicating significantly different points. Asterisks indicate a significant difference between conditions at a given time point. Error bars are within-subjects error bars for panel a and model error bars for panel B. CxT: condition × time interaction.

For focus, a significant condition × time interaction (*p* < 0.001) was observed (Figure 3a). Follow-up indicated that, in the RTD condition, focus was lower at Pre2 (*p* = 0.004; 29% difference) and higher at Post2 (*p* = 0.03; 20% difference), as compared to Pre1. No significant differences between Pre1 and Post1 (*p* = 0.43) or Pre1 and Post3 (*p* = 0.08) were present. In PL, focus was lower at Pre2 (*p* = 0.002; 24% difference) than Pre1 but did not differ from Pre1 at other timepoints (*p* = 0.28 to 0.60). Focus did not differ between conditions at Pre1 (*p* = 0.49), Pre2 (*p* = 0.28), or Post1 (*p* = 0.60), but was higher in RTD than PL at Post2 (*p* = 0.01; 24% difference) and Post3 (*p* = 0.02; 18% difference).

For concentration, a significant condition × time interaction (*p* < 0.001) was observed (Figure 3b). Follow-up indicated that, in the RTD condition, concentration was lower at Pre2 (*p* = 0.03; 20% difference) and higher at Post2 (*p* = 0.04; 22% difference), as compared to Pre1, but did not differ between Pre1 and Post1 (*p* = 0.30) or Post3 (*p* = 0.12). In PL, concentration did not differ from Pre1 at any subsequent timepoints. Concentration did not differ between conditions at Pre1 (*p* = 0.94), Pre2 (*p* = 0.66), or Post1 (*p* = 0.96), but was higher in RTD than PL at Post2 (*p* = 0.02; 24% difference) and Post3 (*p* = 0.02; 20% difference).

For alertness, a significant condition × time interaction (*p* = 0.002) was observed (Figure 3c). Follow-up indicated that, in the RTD condition, alertness was lower at Pre2 (*p* = 0.001; 40% difference) and higher at Post2 (*p* = 0.03; 25% difference), as compared to Pre1, without differences between Pre1 and Post1 (*p* = 0.18) or Post3 (*p* = 0.89). In PL, alertness was lower at Pre2 (*p* = 0.004; 25% difference) and higher at Post1 (*p* = 0.04; 16% difference) as compared to Pre1, without differences between Pre1 and Post2 (*p* = 0.89) or Post3 (*p* = 0.95). Alertness was higher in RTD than PL at Post2 (*p* = 0.02; 26% difference) and Post3 (*p* = 0.02; 20% difference) but did not differ between conditions at Pre1 (*p* = 0.82), Pre2 (*p* = 0.19), or Post1 (*p* = 0.89).

For mood, a significant condition × time interaction (*p=*0.02) was observed (Figure 3d). However, follow-up did not reveal differences between Pre1 and subsequent time points for RTD (*p* = 0.05 to 0.21) or PL (*p* = 0.16 to 0.58). Similarly, mood ratings did not significantly differ between conditions at any time point (*p* = 0.05 to 0.99) after correction for multiple comparisons.

### 3.4. Side effects

No serious side effects were reported. Overall, side effects were reported in six (10.7%) of the 56 total visits. Of these, two reports were for the PL condition and included headache (*n* = 1) and increased energy (*n* = 1). For the RTD condition, reported side effects included headache (*n* = 1), jittery feeling (*n* = 1), mild acid reflux (*n* = 1), and changes in urine color (*n* = 1).

### 3.5. Blinding efficacy

Nineteen out of 28 participants (~68%) were able to correctly identify that they had consumed the RTD beverage. Twenty out of 28 participants (~71%) were able to correctly identify that they had consumed PL.

## 4. Discussion

The purpose of the present study was to determine the acute effect of an RTD thermogenic beverage formulation on REE, RQ, hemodynamic function, and subjective ratings of energy, focus, concentration, alertness, and mood. The primary findings were that acute consumption of the RTD beverage significantly elevated REE compared to PL without negatively affecting hemodynamic variables. Though REE significantly increased post-ingestion, no statistical interactions were present for RQ, suggesting the increase in REE was due to increases in both carbohydrate and fat oxidation. In addition, the RTD beverage increased subjective measures of energy, focus, concentration, and alertness relative to PL.

Multi-ingredient thermogenic products are commonly sought after by individuals aiming to lose body fat and increase energy. These supplements typically contain a blend of active ingredients suggested to increase energy expenditure and promote lipolysis. The RTD beverage employed in the present analysis contained 180 mg per serving of caffeine coming from a blend of caffeine anhydrous, green tea extract, and guarana seed extract (Table 3), with participants consuming two servings for a total of 360 mg caffeine. In addition, L-carnitine and inositol were included, possibly due to their potential role in modulating fat oxidation, though findings are mixed [32]. Caffeine is a β-agonist that elevates REE by stimulating the β_2_ and β_3_ adrenergic receptors [16]. Caffeine also increases REE through the activation of cAMP, by inhibiting transcellular phosphodiesterase, followed by an increase in circulating epinephrine [16]. Green tea extract may exert its effects through catechin polyphenols, such as epigallocatechin gallate, epigallocatechin, epicatechin gallate, and epicatechin [32]. Guarana is a natural plant source of caffeine that contains similar properties to caffeine anhydrous [32]. While these ingredients may provide thermogenic benefits individually, the increase in REE in the present study can most likely be attributed to the ingredients working in concert to promote thermogenesis. Indeed, in the present study, REE increased 12.4% and 9.2% at 35–50- and 85–100-minutes post-ingestion, respectively, as compared to baseline (Figure 1a). Corresponding percent differences as compared to the PL condition were 5.2% and 5.5% at these time points. While previous investigations have demonstrated increases of REE ranging from 8% to 29% post-ingestion of a thermogenic supplement, differences could be attributed to the dosages used, combination of ingredients, and the concentrations of individual ingredients [12–16]. For instance, Wilborn et al. [16] found that a commercially available thermogenic supplement increased REE by 17.3%, 19.6%, and 15.3% at the 1 h-, 2 h-, and 3-h time points, respectively, compared to baseline. The supplement studied by the researchers consisted of 300 mg of caffeine and 690 mg of a proprietary blend of chocamine, green tea extract, yohimbine-HCL, L-taurine, and L-tyrosine. Hoffman et al. [8] determined that a weight loss supplement consisting of anhydrous caffeine, synephrine, tetradecylthioacetic acid, yerba mate extract, methylphenylethylamine, yohimbine, and hordenine increased REE by 29% in a 3-hour post-ingestion window. Additionally, Outlaw et al. [12] reported that a commercially available thermogenic supplement increased REE 8% for at least four hours post-ingestion in a cohort of physically active males and females. The supplement used in their study had 340 mg of caffeine coming from a proprietary blend of caffeine anhydrous, guarana, yerba mate, and green tea extract, relatively similar to the one used in the present analysis. Finally, most recently, Campbell et al. [13] noted a 9.0%, 11.5%, and 9.7% increase in REE at 60-, 120- and 180-minutes post-ingestion of a thermogenic supplement consisting of caffeine, capsaicinoids, and L-carnitine. As such, the unique combination of active ingredients, concentration of individual ingredients, and potentially the mode of ingestion (capsule versus liquid) may have contributed to the differing effects of these supplements on REE. Nonetheless, the current study agrees with the aforementioned analyses in that it demonstrated a significant increase in REE that was sustained at least 100-minutes post-ingestion. Importantly, effects of acute consumption may differ from chronic use, and as such, it is important to discuss the potential utility of RTD for habitual use. Two analyses have reported maintenance of elevated REE and/or limited changes in safety biomarkers after 28 days of supplementation with a thermogenic supplement [17,23]. Therefore, while chronic use of the thermogenic beverage utilized in the present study was not addressed, it is possible that the observed elevations in REE would still be present after chronic supplementation. However, further research is needed to elucidate the utility of this specific RTD for increasing thermogenesis chronically, as well as to evaluate the safety of continued consumption. The elevation of REE relative to baseline in the PL condition can likely be attributed to the ingestion of 24 fluid ounces (710 ml) of a cold beverage (4°C). Indeed, ingestion of 500 ml of cold water has previously been shown to elevate energy expenditure by 2.9% at 90 minutes following ingestion [33]. In the present study, the change in REE relative to baseline in the PL condition was nearly identical at a similar time interval, with a non-statistically-significant 3.0% elevation at 85–100 minutes following ingestion. The increase relative to baseline at the earlier time interval, 35–50 minutes, was higher at 6.4% (*p* < 0.05), indicating that, as expected, the thermogenic effect of ingesting cold fluid diminishes as the fluids are assimilated. As both the RTD and PL beverages were kept in the same refrigerator and were isovolumetric, the observed significant differences between conditions for post-ingestion REE can be attributed to the active ingredients in the RTD beverage rather than the temperature or volume of fluid ingested.

A secondary purpose of this investigation was to determine the impact of a RTD beverage on hemodynamic function. No statistical interactions were present for HR; however, a main effect of condition indicated lower HR in RTD as compared to PL (3.0 ± 0.9 bpm; *p* = 0.003). Though interesting, the condition main effect includes the pre-beverage ingestion timepoint for HR. Therefore, without a condition x time interaction, we cannot conclude the lower HR in the RTD condition compared to P was not partially due to participants presenting with a slightly lower HR prior to beverage ingestion. Additionally, a time main effect and follow up tests also revealed a lower HR at 35–50 minutes post-ingestion relative to baseline (−2.3 ± 0.8 bpm; *p* = 0.02) and 85–100 min (−3.7 ± 1.3 bpm; *p* = 0.02) (Figure 1d). Several of the participants had not experienced a REE assessment utilizing a ventilated hood prior to their participation in this study; therefore, we speculate participants felt more relaxed at the 35–50 minutes post-ingestion time point given that the initial REE assessment had already been completed. This, coupled with the fact that hemodynamic assessments were performed after the supine rest periods could have contributed to the participants feeling more calm and less anxious at this specific time point. Additionally, the sex main effect revealed that females had a higher HR than males (8.3 ± 3.2 bpm; *p* = 0.02), irrespective of condition or time. This could have been a result of the training status of participants (i.e., within RT and NRT, the activity level of each participant may have varied), random differences, or potentially factors related to relative caffeine doses as later discussed. For BP, a sex main effect indicated higher SBP in males as compared to females (10.7 ± 2.5 mmHg; *p* < 0.001) (Figure 1d). Men are generally at greater risk of cardiovascular and renal disease than age-matched, premenopausal women [34]. Indeed, prior data applying the technique of 24-hour ambulatory BP monitoring have demonstrated that BP is higher in men than in women at similar ages [34]. These data likely explain males presenting with a higher SBP than females in the current analysis. Furthermore, condition main effects revealed significantly higher SBP (3.5 ± 1.1 mm Hg; *p* = 0.003) and DBP (3.5 ± 0.9 mm Hg; *p* < 0.001) in the RTD condition as compared to PL, irrespective of time. However, all values remained within normal ranges. Still, while hemodynamic values did not fall outside of normal ranges, the magnitude of change should also be considered. For example, a recent meta-analysis found that every 10 mmHg increase of SBP and 5 mmHg increase of DBP was reported to be associated with an increased risk of cardiovascular events, coronary heart disease, stroke, and all-cause mortality [35]. Therefore, though acute consumption of the RTD beverage did not cause a ≥10 mmHg increase of SBP or a ≥5 mmHg increase of DBP, it is possible chronic consumption could produce changes in SBP and DBP commensurate with what has been associated with a greater risk for the aforementioned events and conditions. Therefore, continued research is needed before definitive conclusions can be made regarding the safety of chronic consumption. While prior investigations have indicated that chronic consumption (up to ~1 month) of a multi-ingredient thermogenic supplement has minimal effects on hemodynamic metrics, others have demonstrated acute elevations in BP following ingestion [8,13–15,17,23]. Hoffman et al. [8] and Campbell et al. [15] both reported significant increases in BP for three hours post-ingestion leading them to suggest that caution may be warranted for individuals with cardiovascular disease before using thermogenic supplements. It should be noted that the hemodynamic response to these supplements is likely affected by the number of active ingredients in the supplement. That is, both aforementioned investigations utilized thermogenic supplements that contain a larger number of active ingredients than the RTD beverage used in the present study. While caution should always be warranted when utilizing energy products and weight loss supplements – and it cannot be ruled out that HR or BP could rise to adverse levels – the RTD beverage employed in the current study appears to be safe for acute consumption in healthy college-aged individuals.

Finally, in agreement with prior investigations, the RTD beverage influenced subjective outcomes assessed by VAS [7,12]. Statistical interactions demonstrated that energy, focus, concentration, and alertness were significantly (*p* < 0.05) greater at 50-minutes post-ingestion as compared to baseline measures (Figure 2a; Figure 3a-c). Additionally, these same metrics were all significantly higher in RTD than PL at 50- and 100-minutes post-ingestion. In addition to the previously discussed ingredients present in the RTD beverage, L-Tyrosine and Taurine are also relevant components. Taurine has been suggested to improve mental focus and concentration, while L-Tyrosine prevents depletion of catecholamines, ameliorating declines in cognition with acute stress [32]. With that being said, of the active ingredients included in the RTD beverage, caffeine is the most studied and regarded as the main contributor to the positive changes reported in the present study [12]. Caffeine is believed to exert its effects on the central nervous system via the antagonism of adenosine receptors, leading to increases in the concentration of certain neurotransmitters such as serotonin, dopamine, acetylcholine, norepinephrine, and glutamate [21]. This is purported to result in positive effects on mood, vigilance, focus, and alertness in most, but not all, individuals [21]. Nonetheless, the aforementioned subjective findings could have potentially been influenced by the Hawthorne effect [36]. That is, the participants may have altered their VAS responses as a motivational reaction to the interest, care, or attention received throughout the study visit [36]. In other words, it is possible when given the VAS to record their subjective values, participants responded with what they thought was the desired response of the researchers [36]. Therefore, we cannot rule this out as a potential confounding factor, although the randomized, double-blind design likely minimized these effects.

A unique characteristic of this study was the strict inclusion/exclusion criteria implemented when recruiting participants. Though limited, prior data demonstrated the potential for training status to affect the metabolic response to caffeine [19,20]. Indeed, Leblanc et al. [19] noted that when 8 endurance-trained (marathon runners and cross-country skiers who engaged regularly in high levels of physical activity) and 8 sedentary males ingested 4 mg/kg of caffeine, a larger increase in REE was observed in the endurance-trained group. Additionally, significant differences were found between the trained and sedentary subjects in plasma free fatty acids and RQ at 20- and 40-minutes post-ingestion. Poehlman et al. [20] also evaluated the effect of caffeine on REE in 14 endurance-trained (long-distance runners, participating in active training for at least 3 years and running 100–160 km/wk) and 10 inactive male individuals. Unlike the previously discussed study, following consumption of 300 mg of caffeine, inactive subjects presented with a larger increase (*p* < 0.05) in REE than the endurance-trained subjects. Given the mixed findings regarding the effects of training status on the metabolic response to caffeine, as well as the lack of information specific to resistance-trained populations, we deemed it necessary for further research be conducted to elucidate this potential relationship. Out of the 28 males and females that participated in our study, 14 were RT (males = 7, females = 7) and 14 were NRT (males = 7, females = 7). In addition, we utilized endurance cutoffs (≤60 minutes of low-intensity steady-state endurance exercise per week and≤30 minutes of HIIT per week) to exclude “endurance trained” individuals due to the results of the aforementioned investigations. This allowed us to not only examine the influence of the RTD beverage on metabolic, hemodynamic, and subjective variables in the sample as a whole but also between RT and NRT individuals. Ultimately, no significant differences in the metabolic responses based on RT status were identified. However, a sex × RT status interaction was observed for energy ratings, with higher values in male NRT participants as compared to male RT participants (*p* = 0.03) (Figure 2b). In addition, DBP was higher in NRT individuals as compared to RT individuals (4.2 ± 1.9 mmHg; *p* = 0.04) (Figure 1e). While these findings are limited by the relatively small sample size within each sex and RT status combination, self-reported caffeine intake was lower in male NRT (301.4 ± 107.3 mg) and all NRT (268.2 ± 85.3 mg) subjects compared to male RT (435.7 ± 121.5 mg) and all RT (335.7 ± 137.9 mg) subjects **(Supplementary Material 1)**. Additionally, self-reported caffeine intake was higher in males (368.6 ± 130.3 mg/d; 4.42 ± 1.56 mg/kg/d) than females (235.4 ± 46.8 mg/d; 3.80 ± 0.90 mg/kg/d) within our sample (Table 2), with males reporting a caffeine intake almost identical to the dosage employed in the present study (360 mg). Caffeine habituation and the presence of certain genotype variations have been reported as potential factors influencing the inter-individual variation in the response to caffeine [37]. Therefore, the lower habitual caffeine intake could have led to the NRT individuals being affected to a greater extent by the caffeine content in the RTD beverage, potentially masking differential effects of RT status per se. In addition, Harty et al. [18] suggested that RT males and females may respond differently to acute caffeine supplementation given differences in body size, body composition, and hormonal milieu [38]. Future research may need to individualize caffeine dosages between individuals given these potential factors, and additional investigations may clarify the influence RT status, habitual caffeine intake, and other factors may have on the metabolic response to acute caffeine ingestion. Finally, the differences in body size between the males (height: 175.0 ± 7.5 cm; weight: 83.8 ± 7.2 kg) and females (height: 163.7 ± 5.4 cm; weight: 62.9 ± 8.1 kg) recruited in this study are noteworthy. Given that an absolute dosage of caffeine was used in the present study, this could have contributed to: 1) smaller individuals being influenced more by the beverage, 2) those with a lower reported daily caffeine intake being influenced more by the beverage, or 3) a combination of both. For instance, as mentioned previously, the self-reported caffeine intake was higher for males as compared to females in the current analysis; while the mean relative caffeine dose of 4.32 mg/kg BM in the RTD condition for males was similar to the habitual mean intake of 4.42 mg/kg, the mean relative caffeine dose of 5.81 mg/kg BM for females was higher than the habitual mean intake of 3.80 mg/kg. Therefore, females ingested a larger relative dose of caffeine compared to the males and their own habitual intake in the present study. Importantly, the absolute dose of caffeine was used due to its presence in the commercially available product and to maximize generalizability to consumption in real-world settings.

The present study is not without limitations. Given the strict inclusion/exclusion criteria, the generalizability may be limited to other populations such as endurance-trained individuals or older adults. Therefore, results may differ in populations of a different age, activity level, or health status. In addition, since the current study only included habitual caffeine consumers, the results cannot be extrapolated to non-consumers. Furthermore, we utilized self-reported caffeine intake to determine habitual consumers, which may not be as reliable as more detailed assessment methods. That is, large variability in the caffeine content of commonly consumed beverages have been reported, and as such, an objective measure (e.g. caffeine or metabolite levels) might be more suitable to report caffeine intake [21]. As mentioned previously, the RTD beverage was administered at an absolute dose, so results may have been different if relative doses were provided. Since we did not observe participants beyond their laboratory visit, we are unable to report subsequent effects or side effects that occurred after the laboratory visit. Furthermore, given equipment limitations, we did not monitor the QT-interval or similar indicators to assess potential cardiovascular changes due to the beverage consumption, which could have provided additional data to either support or refute the safety of the thermogenic beverage. The percentage of participants correctly identifying the ingested beverage was slightly higher than reported by some previous double-blind, placebo-controlled investigations which examined caffeine-containing products [37,39]. Potentially, the participants may have been able to differentiate between conditions based off the characteristically bitter taste of caffeine [39]. Finally, though menstrual cycle data was recorded, visits were not scheduled around phases of participants’ menstrual cycle.

## 5. Conclusion

Caffeine-containing products such as pre-workout supplements, energy drinks, and weight loss supplements continue to increase in popularity and are common sources of caffeine in peoples’ diets. It is imperative randomized clinical trials be conducted to determine the safety and efficacy of caffeine-containing thermogenic products. In this regard, no serious side effects were reported after consuming the RTD beverage. Within the context of the present study (male and female resistance-trained and non-resistance-trained college-aged individuals), acute ingestion of an RTD beverage formulation significantly increased REE and feelings of energy, focus, concentration, and alertness without producing negative effects on hemodynamic variables. While the supplement used in the current study could therefore be viewed as safe for acute consumption and effective for increasing metabolic rate for at least 100 minutes post-ingestion, future research is needed to determine effects of chronic consumption. That is, acute increases in energy expenditure or lipolysis may not equate to meaningful reductions in body weight or fat mass over time [40]. Therefore, lifestyle interventions aimed at improving health status and body composition should include a combination of diet and exercise therapies as opposed to solely diet-related interventions (e.g., thermogenic products) to facilitate more effective outcomes [10]. Consumers interested in thermogenic products should seek out evidence demonstrating their safety and effectiveness before consuming. Additionally, future research should continue to document the safety and efficacy of popular commercially available products for acute and chronic consumption.

## Supplementary Material

Supplemental Material

## Data Availability

Data may be available from corresponding author upon reasonable request, pending relevant approvals and institutional review.

## List of abbreviations

ANOVA: analysis of variance
BP: blood pressure
cAMP: 3,5-cyclic-adenosine monophosphate
DBP: diastolic blood pressure
HIIT: high-intensity interval training
HR: heart rate
NRT: non-resistance trained
PL: placebo
REE: resting energy expenditure
RQ: respiratory quotient
RT: resistance trained
RTD: ready-to-drink
SBP: systolic blood pressure
VAS: visual analog scales
