# Supplementary material for: Effects of a ready-to-drink thermogenic beverage on resting energy expenditure, hemodynamic function, and subjective outcomes
Source: J Int Soc Sports Nutr. 2023 May 10;20(1):2211958. doi: 10.1080/15502783.2023.2211958 (PMC10173796; doi:10.1080/15502783.2023.2211958)
Supplement: Supplemental Material [file RSSN_A_2211958_SM6074.zip › Supplemetary/Supplementary Table 1.docx]

|  | **F (n=14)** | | **M (n=14)** | | **NRT (n=14)** | | **RT (n=14)** | |
| --- | --- | --- | --- | --- | --- | --- | --- | --- |
|  | mean | sd | mean | sd | mean | sd | mean | sd |
| Age (y) | 22.3 | 3.0 | 24.3 | 4.4 | 22.7 | 4.0 | 23.9 | 3.8 |
| Height (cm) | 163.7 | 5.4 | 175.0 | 7.5 | 170.3 | 8.5 | 168.4 | 9.0 |
| Weight (kg) | 62.9 | 8.1 | 83.8 | 7.2 | 72.3 | 13.6 | 74.4 | 13.0 |
| BMI (kg/m2) | 23.5 | 2.9 | 27.4 | 2.5 | 24.8 | 3.5 | 26.1 | 3.2 |
| FFMI (kg/m2) | 16.6 | 1.3 | 20.6 | 1.7 | 17.9 | 2.4 | 19.3 | 2.6 |
| Body fat (%) | 28.8 | 4.9 | 24.5 | 5.8 | 27.3 | 6.2 | 26.0 | 5.3 |
| RT Experience (years) | 1.6 | 1.8 | 3.7 | 4.9 | 0.0 | 0.0 | 5.3 | 3.8 |
| RT Frequency (days/week) | 2.3 | 2.4 | 2.3 | 2.5 | 0.0 | 0.0 | 4.5 | 0.9 |
| Caffeine Intake (mg/d) | 235.4 | 46.8 | 368.6 | 130.3 | 268.2 | 85.3 | 335.7 | 137.9 |
| Phase Angle (degrees) | 5.5 | 0.5 | 6.5 | 0.7 | 5.8 | 0.7 | 6.2 | 0.9 |
| Waist Circumference (cm) | 88.0 | 7.7 | 92.6 | 5.1 | 91.3 | 7.3 | 89.4 | 6.4 |
| Hip Circumference (cm) | 103.0 | 5.2 | 107.7 | 4.8 | 105.1 | 6.0 | 105.5 | 5.1 |
| Upper Arm Circumference (cm) | 32.5 | 3.2 | 38.2 | 2.6 | 34.5 | 3.6 | 36.2 | 4.4 |
| Forearm Circumference (cm) | 25.6 | 1.6 | 30.0 | 0.8 | 27.3 | 2.6 | 28.3 | 2.5 |
| Thigh Circumference (cm) | 59.3 | 4.8 | 62.6 | 3.6 | 60.1 | 4.7 | 61.8 | 4.3 |
| Calf Circumference (cm) | 36.6 | 2.7 | 39.7 | 1.9 | 38.2 | 3.5 | 38.1 | 1.9 |

**Supplementary Table 1. Participant Characteristics Split by Sex and Resistance Training Status.**

*Note: participant characteristics for the entire sample and sex/RT status subgroups are presented in the main manuscript.*
