# Supplementary material for: Effects of a ready-to-drink thermogenic beverage on resting energy expenditure, hemodynamic function, and subjective outcomes
Source: J Int Soc Sports Nutr. 2023 May 10;20(1):2211958. doi: 10.1080/15502783.2023.2211958 (PMC10173796; doi:10.1080/15502783.2023.2211958)
Supplement: Supplemental Material [file RSSN_A_2211958_SM6074.zip › Supplemetary/Supplementary Table 2.docx]

| **Outcome** | **Effect** | **F** | **Partial Eta Squared** | **p** |
| --- | --- | --- | --- | --- |
| REE | Sex | 69.4 | 0.74 | 0.000 |
|  | RT | 1.8 | 0.07 | 0.189 |
|  | Sex:RT | 0.0 | 0.00 | 0.858 |
|  | Condition | 8.9 | 0.27 | 0.006 |
|  | Sex:Condition | 1.0 | 0.04 | 0.321 |
|  | RT:Condition | 0.6 | 0.03 | 0.439 |
|  | Sex:RT:Condition | 0.1 | 0.00 | 0.813 |
|  | Time | 58.7 | 0.71 | 0.000 |
|  | Sex:Time | 0.3 | 0.01 | 0.695 |
|  | RT:Time | 0.1 | 0.00 | 0.851 |
|  | Sex:RT:Time | 1.0 | 0.04 | 0.358 |
|  | Condition:Time | 18.8 | 0.44 | 0.000 |
|  | Sex:Condition:Time | 0.4 | 0.01 | 0.686 |
|  | RT:Condition:Time | 1.0 | 0.04 | 0.376 |
|  | Sex:RT:Condition:Time | 1.0 | 0.04 | 0.386 |
| RQ | Sex | 3.2 | 0.12 | 0.085 |
|  | RT | 1.0 | 0.04 | 0.322 |
|  | Sex:RT | 1.9 | 0.07 | 0.180 |
|  | Condition | 0.1 | 0.00 | 0.740 |
|  | Sex:Condition | 2.1 | 0.08 | 0.161 |
|  | RT:Condition | 2.1 | 0.08 | 0.158 |
|  | Sex:RT:Condition | 1.1 | 0.05 | 0.294 |
|  | Time | 7.8 | 0.25 | 0.001 |
|  | Sex:Time | 0.3 | 0.01 | 0.727 |
|  | RT:Time | 0.4 | 0.02 | 0.687 |
|  | Sex:RT:Time | 0.6 | 0.02 | 0.557 |
|  | Condition:Time | 1.7 | 0.07 | 0.203 |
|  | Sex:Condition:Time | 0.1 | 0.01 | 0.836 |
|  | RT:Condition:Time | 0.1 | 0.01 | 0.832 |
|  | Sex:RT:Condition:Time | 1.3 | 0.05 | 0.283 |
| HR | Sex | 6.7 | 0.22 | 0.016 |
|  | RT | 0.9 | 0.04 | 0.351 |
|  | Sex:RT | 1.5 | 0.06 | 0.229 |
|  | Condition | 10.9 | 0.31 | 0.003 |
|  | Sex:Condition | 0.6 | 0.02 | 0.446 |
|  | RT:Condition | 0.3 | 0.01 | 0.613 |
|  | Sex:RT:Condition | 1.1 | 0.04 | 0.310 |
|  | Time | 4.8 | 0.17 | 0.022 |
|  | Sex:Time | 0.1 | 0.00 | 0.845 |
|  | RT:Time | 0.1 | 0.01 | 0.809 |
|  | Sex:RT:Time | 1.3 | 0.05 | 0.267 |
|  | Condition:Time | 2.5 | 0.09 | 0.098 |
|  | Sex:Condition:Time | 1.6 | 0.06 | 0.212 |
|  | RT:Condition:Time | 2.0 | 0.08 | 0.152 |
|  | Sex:RT:Condition:Time | 2.4 | 0.09 | 0.112 |
| SBP | Sex | 18.3 | 0.43 | 0.000 |
|  | RT | 0.1 | 0.01 | 0.727 |
|  | Sex:RT | 0.2 | 0.01 | 0.625 |
|  | Condition | 10.6 | 0.31 | 0.003 |
|  | Sex:Condition | 3.3 | 0.12 | 0.081 |
|  | RT:Condition | 0.2 | 0.01 | 0.649 |
|  | Sex:RT:Condition | 0.0 | 0.00 | 0.965 |
|  | Time | 9.9 | 0.29 | 0.000 |
|  | Sex:Time | 0.7 | 0.03 | 0.506 |
|  | RT:Time | 0.1 | 0.00 | 0.936 |
|  | Sex:RT:Time | 1.8 | 0.07 | 0.173 |
|  | Condition:Time | 1.3 | 0.05 | 0.286 |
|  | Sex:Condition:Time | 0.4 | 0.02 | 0.687 |
|  | RT:Condition:Time | 2.8 | 0.10 | 0.072 |
|  | Sex:RT:Condition:Time | 0.7 | 0.03 | 0.506 |
| DBP | Sex | 2.2 | 0.08 | 0.150 |
|  | RT | 4.9 | 0.17 | 0.037 |
|  | Sex:RT | 0.5 | 0.02 | 0.507 |
|  | Condition | 15.5 | 0.39 | 0.001 |
|  | Sex:Condition | 3.3 | 0.12 | 0.083 |
|  | RT:Condition | 0.2 | 0.01 | 0.651 |
|  | Sex:RT:Condition | 0.4 | 0.01 | 0.558 |
|  | Time | 16.5 | 0.41 | 0.000 |
|  | Sex:Time | 1.3 | 0.05 | 0.292 |
|  | RT:Time | 0.2 | 0.01 | 0.831 |
|  | Sex:RT:Time | 0.0 | 0.00 | 0.993 |
|  | Condition:Time | 1.1 | 0.04 | 0.331 |
|  | Sex:Condition:Time | 0.5 | 0.02 | 0.602 |
|  | RT:Condition:Time | 1.0 | 0.04 | 0.363 |
|  | Sex:RT:Condition:Time | 0.7 | 0.03 | 0.469 |
| energy | Sex | 0.0 | 0.00 | 0.997 |
|  | RT | 2.4 | 0.09 | 0.131 |
|  | Sex:RT | 7.5 | 0.24 | 0.011 |
|  | Condition | 2.5 | 0.09 | 0.128 |
|  | Sex:Condition | 0.0 | 0.00 | 0.965 |
|  | RT:Condition | 0.1 | 0.00 | 0.735 |
|  | Sex:RT:Condition | 6.3 | 0.21 | 0.019 |
|  | Time | 16.4 | 0.41 | 0.000 |
|  | Sex:Time | 0.9 | 0.04 | 0.422 |
|  | RT:Time | 1.7 | 0.07 | 0.188 |
|  | Sex:RT:Time | 0.9 | 0.04 | 0.434 |
|  | Condition:Time | 8.3 | 0.26 | 0.000 |
|  | Sex:Condition:Time | 1.3 | 0.05 | 0.297 |
|  | RT:Condition:Time | 0.8 | 0.03 | 0.482 |
|  | Sex:RT:Condition:Time | 0.5 | 0.02 | 0.691 |
| focus | Sex | 0.0 | 0.00 | 0.989 |
|  | RT | 1.0 | 0.04 | 0.324 |
|  | Sex:RT | 3.8 | 0.14 | 0.063 |
|  | Condition | 1.6 | 0.06 | 0.214 |
|  | Sex:Condition | 0.6 | 0.03 | 0.428 |
|  | RT:Condition | 0.0 | 0.00 | 0.973 |
|  | Sex:RT:Condition | 2.2 | 0.08 | 0.148 |
|  | Time | 10.6 | 0.31 | 0.000 |
|  | Sex:Time | 0.4 | 0.02 | 0.688 |
|  | RT:Time | 1.1 | 0.04 | 0.358 |
|  | Sex:RT:Time | 0.5 | 0.02 | 0.604 |
|  | Condition:Time | 7.4 | 0.24 | 0.000 |
|  | Sex:Condition:Time | 1.3 | 0.05 | 0.294 |
|  | RT:Condition:Time | 1.1 | 0.04 | 0.349 |
|  | Sex:RT:Condition:Time | 0.8 | 0.03 | 0.490 |
| concentration | Sex | 0.3 | 0.01 | 0.604 |
|  | RT | 0.3 | 0.01 | 0.574 |
|  | Sex:RT | 3.4 | 0.12 | 0.078 |
|  | Condition | 2.6 | 0.10 | 0.121 |
|  | Sex:Condition | 0.4 | 0.01 | 0.557 |
|  | RT:Condition | 0.1 | 0.00 | 0.823 |
|  | Sex:RT:Condition | 1.2 | 0.05 | 0.279 |
|  | Time | 7.7 | 0.24 | 0.001 |
|  | Sex:Time | 1.6 | 0.06 | 0.204 |
|  | RT:Time | 0.4 | 0.01 | 0.694 |
|  | Sex:RT:Time | 1.4 | 0.05 | 0.263 |
|  | Condition:Time | 6.6 | 0.22 | 0.001 |
|  | Sex:Condition:Time | 0.8 | 0.03 | 0.469 |
|  | RT:Condition:Time | 1.8 | 0.07 | 0.166 |
|  | Sex:RT:Condition:Time | 0.6 | 0.03 | 0.592 |
| alertness | Sex | 0.7 | 0.03 | 0.427 |
|  | RT | 1.6 | 0.06 | 0.225 |
|  | Sex:RT | 2.6 | 0.10 | 0.119 |
|  | Condition | 3.0 | 0.11 | 0.095 |
|  | Sex:Condition | 0.5 | 0.02 | 0.508 |
|  | RT:Condition | 0.4 | 0.02 | 0.519 |
|  | Sex:RT:Condition | 1.2 | 0.05 | 0.293 |
|  | Time | 15.1 | 0.39 | 0.000 |
|  | Sex:Time | 1.5 | 0.06 | 0.223 |
|  | RT:Time | 1.0 | 0.04 | 0.368 |
|  | Sex:RT:Time | 0.8 | 0.03 | 0.475 |
|  | Condition:Time | 5.5 | 0.19 | 0.002 |
|  | Sex:Condition:Time | 2.2 | 0.08 | 0.099 |
|  | RT:Condition:Time | 1.0 | 0.04 | 0.393 |
|  | Sex:RT:Condition:Time | 0.9 | 0.03 | 0.471 |
| mood | Sex | 0.0 | 0.00 | 0.875 |
|  | RT | 0.2 | 0.01 | 0.623 |
|  | Sex:RT | 3.1 | 0.11 | 0.091 |
|  | Condition | 2.4 | 0.09 | 0.133 |
|  | Sex:Condition | 0.5 | 0.02 | 0.469 |
|  | RT:Condition | 0.0 | 0.00 | 0.907 |
|  | Sex:RT:Condition | 0.0 | 0.00 | 0.912 |
|  | Time | 7.0 | 0.23 | 0.000 |
|  | Sex:Time | 0.4 | 0.02 | 0.744 |
|  | RT:Time | 0.5 | 0.02 | 0.654 |
|  | Sex:RT:Time | 0.6 | 0.03 | 0.595 |
|  | Condition:Time | 3.6 | 0.13 | 0.017 |
|  | Sex:Condition:Time | 0.9 | 0.03 | 0.470 |
|  | RT:Condition:Time | 1.7 | 0.07 | 0.166 |
|  | Sex:RT:Condition:Time | 2.2 | 0.08 | 0.098 |

**Supplementary Table 2. Analysis of Variance Results.**
